# The role of exosomal long non-coding RNAs in cancer drug resistance

**DOI:** 10.20517/cdr.2019.74

**Published:** 2019-12-19

**Authors:** Mireia Cruz De los Santos, Mihnea P. Dragomir, George A. Calin

**Affiliations:** 1 Department of Experimental Therapeutics, The University of Texas MD Anderson Cancer Center, Houston, TX 77054, USA; 2 Research Center for Functional Genomics, Biomedicine and Translational Medicine, Iuliu Hatieganu University of Medicine and Pharmacy, Cluj-Napoca 40015, Romania; 3 Department of Surgery, Fundeni Clinical Hospital, Carol Davila University of Medicine and Pharmacy, Bucharest 022328, Romania; 4 Center for RNA Interference and Non-coding RNAs, The University of Texas MD Anderson Cancer Center, Houston, TX 77054, USA

**Keywords:** Tumor microenvironment, extracellular vesicles, exosomes, non-coding RNA, long non-coding RNA, drug resistance

## Abstract

One of the major challenges in oncology is drug resistance, which triggers relapse and shortens patients’ survival. In order to promote drug desensitization, cancer cells require the establishment of an ideal tumor microenvironment that accomplishes specific conditions. To achieve this objective, cellular communication is a key factor. Classically, cells were believed to restrictively communicate by ligand-receptor binding, physical cell-to-cell interactions and synapses. Nevertheless, the crosstalk between tumor cells and stroma cells has also been recently reported to be mediated through exosomes, the smallest extracellular vesicles, which transport a plethora of functionally active molecules, such as: proteins, lipids, messenger RNA, DNA, microRNA or long non-coding RNA (lncRNAs). LncRNAs are RNA molecules greater than 200 base pairs that are deregulated in cancer and other diseases. Exosomal lncRNAs are highly stable and can be found in several body fluids, being considered potential biomarkers for tumor liquid biopsy. Exosomal lncRNAs promote angiogenesis, cell proliferation and drug resistance. The role of exosomal lncRNAs in drug resistance affects the main treatment strategies in oncology: chemotherapy, targeted therapy, hormone therapy and immunotherapy. Overall, knowing the molecular mechanisms by which exosomal lncRNA induce pharmacologic resistance could improve further drug development and identify drug resistance biomarkers.

## INTRODUCTION

Cancer represents the second preeminent cause of death worldwide, being a noticeable public health issue and a huge socioeconomic burden^[1]^. Specifically, lung and breast cancers are the malignancies with the highest incidence and are the most lethal cancers in men and women, respectively. Although in recent decades life expectancy for certain cancers has risen due to early detection and treatment, cancer mortality is still a significant problem in oncology^[2]^. The main trigger contributing to increased cancer mortality is the tumoral ability to become therapy-resistant through genetic^[3]^ or epigenetic^[4]^ mechanisms. Drug invulnerability is divided in two existing types: intrinsic and acquired. Intrinsic multidrug resistance consists in the inherent potential of cancer cells to be resistant before any treatment is administered. This resistance subtype was believed to be the one responsible of drug resistance in most cases. Nevertheless, cancer treatment induces a natural selection of competent cellular populations generating what is called, acquired therapy resistance. Nowadays, drug resistance is considered a combined, complex, and multistep process^[5]^. Despite the fact that drug resistance is intensely researched, the molecular mechanisms underlying cancer cells desensitization are still undefined. Tumor microenvironment and lncRNAs have been shown to play a leading function in pharmacological resistance. This review aims to compile the current knowledge in this area in order to enlighten future directions and perspectives in cancer therapy development.

## TUMOR MICROENVIRONMENT AND THERAPY RESISTANCE

The tumor is a complex system composed of cancer and normal cells (tumor microenvironment), the last of which actively participate in tumorigenesis, helping the tumor to acquire hallmark capabilities^[6,7]^. The tumor microenvironment is composed of different cell types like, endothelial cells, adipocytes, immune cells, fibroblasts, *etc.*, surrounded by soluble factors and an extracellular matrix (ECM) which exhibits specific physicochemical properties^[8,9]^.

Researchers have, indeed, highlighted tumor microenvironment’s function in drug resistance, leading to progression, invasion and metastasis^[6,7]^. In breast cancer^[10]^, it has been demonstrated that non-neoplastic stroma cells are subject to gene expression alterations promoted by cancer cells. Hence, the specific epigenetic marks found on tumor microenvironment cells are predictive of prognosis and should be also considered as potential therapeutic targets^[11]^. In addition, Vemurafenib treatment, a kinase-inhibitor targeted drug, was shown to change the secretome in melanoma and lung adenocarcinoma, enhancing the formation of a microenvironment that selectively supports drug resistant clones^[12]^. Altogether these data exemplify the tight relation between tumor microenvironment and cancer cells resistance acquisition. Nevertheless, it has been demonstrated that reversion of pharmacological-resistant phenotypes is possible, evidencing the importance of epigenetics alterations and, microenvironment in drug resistance^[12–14]^. Thus, cancer drug desensitization cannot be understood by only targeting the tumor cell itself but taking into account the surrounding tumor-associated stroma and microenvironment.

Tumors do not only shape the development of a local microenvironment, but also set up the appropriate conditions at distant organs in order to ensure their survival before they migrate to distant sites. These sites are known as pre-metastatic niches (PMNs). One of the main factors regarding metastasis is the generation of these preordained areas which enable the cells to succeed in their migration ^[8,9,15,16]^. In addition, drug-resistant clones in the primary tumor are more competent to undergo epithelial-mesenchymal transition (EMT) and present higher probabilities to reach a favorable PMN^[5]^. In this context, an essential question emerges: How is it possible for the tumor to modify distant or surrounding cells in order to create future metastatic niches or to induce drug-resistance? Cellular communication is the key point. Traditionally, the most acquainted mechanisms involved in cellular communication were ligand-receptor events (autocrine, paracrine and endocrine)^[17]^, synapses (neuronal and neuromuscular) and cell-to-cell physical interactions^[18]^. In recent years, a new system of cellular communication has been elucidated: it has been demonstrated that the coordinated action of factors secreted by the tumor, such as extracellular vesicles (EVs) are crucial for cellular crosstalk and pharmacological-resistance promotion. Therefore, in the following sections the mechanisms involving EVs in drug desensitization will be described.

## EXTRACELLULAR VESICLES

EVs are double-layered lipid extracellular constructs, which play a crucial role in cellular communication and can be secreted by all cell types. EVs are diverse, being composed of a heterogeneous group of membranous structures. According to the mechanism of production and size, EVs are divided into (1) apoptotic bodies; (2) microvesicles; and (3) exosomes. Apoptotic bodies range from 500 nm to 2000 nm and are the result of membrane blebbing of apoptotic cells. Microvesicles (50-1000 nm) are generated by budding of the cell plasma membrane, and exosomes (50-150 nm) are produced within multivesicular bodies (MVBs) from the endolyososomal system^[19–21]^.

EVs mediate cellular crosstalk by transferring their cargoes, such as lipids, proteins, transmembrane receptors, messenger RNAs (mRNAs) and non-coding RNAs (ncRNAs), into recipient cell. EVs’ biogenesis and cargo composition is dependent on its particular releasing cell-type and varies between physiologic or pathologic conditions^[22]^. Emerging data defend the regulatory function of EVs and the molecular cargo is considered the active component of exosomes^[23]^. Multiple research groups have proven the importance of EVs in cancer cell dissemination and tumor progression. In fact, EVs increase cell motility and local invasiveness by changing the composition of the ECM^[24]^, promoting drug resistance^[25]^ and allowing the generation of invadopodia, membrane projections with enzymatic activity^[26]^. Moreover, EVs exert a crucial function in distant metastasis establishing PMNs at distant sites^[26–28]^. In cancer biology, exosomes were proven to be the EVs with the most important regulatory function and are the most in depth studied extracellular construct linked to cancer^[29]^. Consequently, we will be further focusing on exosomes as crosstalk vectors.

## EXOSOMES: CELL COMMUNICATION, BIOGENESIS AND DRUG RESISTANCE

### Exosomes mediate cellular communication

Tumor microenvironment exerts an essential function in tumor promotion, metastasis and drug resistance. In this regard, exosomes act as shipping packages, delivering functional molecules to target cells. Tumor-derived exosomes have been shown to enhance cancer progression by targeting immunocytes. For example, Chen *et al.*^[30]^ reported that ovarian cancer-derived exosomes transferred miR-940 to macrophages switching their phenotype to an M2-like. This anti-inflammatory phenotype results in metastasis and high mortality rates. Simultaneously, exosome communication also occurs in the opposite direction: immune cells release exosomes that reach cancer cells. Exosomes secreted by natural killer cells, enriched with perforin and Fas L, selectively kill melanoma cells without harming non-neoplastic kidney cells^[31]^.

Apart from immune cells, exosomes are also reached and secreted by distinct stromal cells, including fibroblasts. In cancer, local fibroblasts are turned into cancer-associated fibroblasts (CAFs) which reinforce the formation of a pro-tumoral microenvironment^[32]^. This phenotypic transformation can be achieved through the exosomal transmission of hTERT mRNA secreted by several cancer cells^[33]^. Furthermore, CAF-derived exosomes have been evidenced to remodel tumor cell metabolism, boosting glycolysis and inhibiting mitochondrial oxidative phosphorylation^[34]^. Thus, exosomes represent a dynamic system for cellular communication in pathologic and physiologic conditions than can modulate gene expression.

Exosomes can be found in diverse body fluids and can act in an endocrine, paracrine or autocrine way. In order to target specific cell populations, exosomes differentially express transmembrane molecules on their surface that will be activated once the vesicle has been released from the donor cell. Some of these molecules are CD63, CD9, CD81 and CD82 tetraspanins, major histocompatibility complex (MHC), flotillin and other receptors^[20,23]^. As soon as the exosome has reached the target cell, it can stay there attached to membrane receptors modulating intracellular pathways^[35,36]^ or can be ingested via endocytosis^[20]^.

### Exosome biogenesis

**Figure 1.**
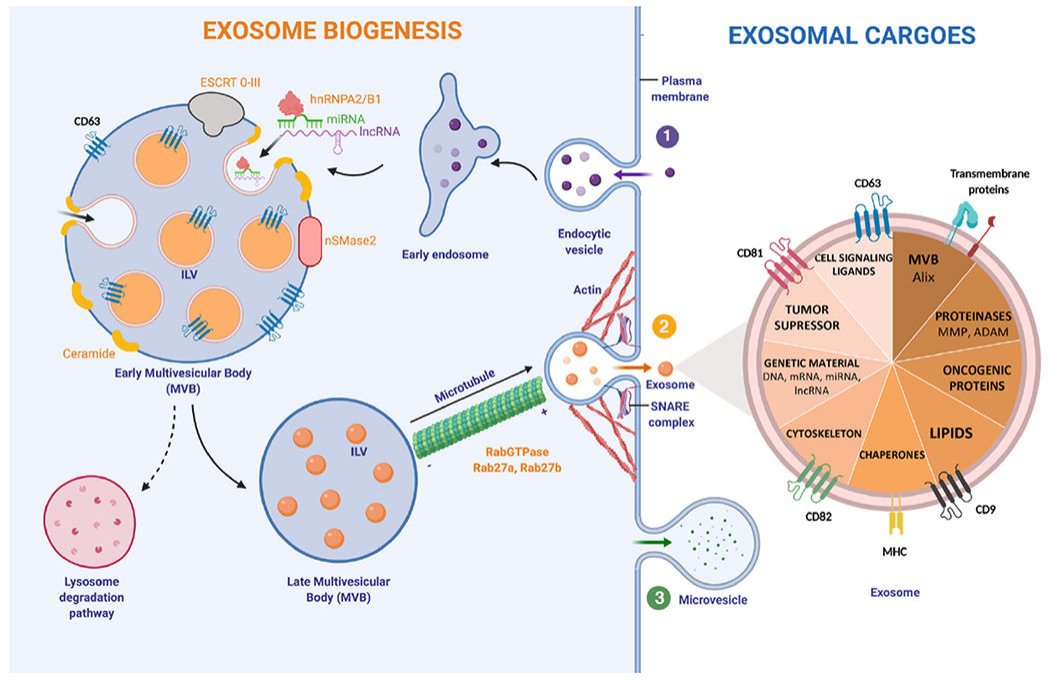
Exosome biogenesis and major exosomal cargoes. (1) Exosome biogenesis starts with the formation of an endosome from the plasma membrane. The successive inward budding of the endocytic vesicle generates ILV, which will generate a MVB. This step can be controled by two pathways; ESCRT-dependent (ESCRT 0-III complex) or ESCRT-independent (nSMase2). Exosomal cargoes approach the MVB membrane in order to be loaded into exosomes. Specifically, RNA-binding molecules bind to miRNAs (hnRNPA2/B1) and lncRNAs to sort them. Other molecules involved in exosomal cargo loading are tetraspanins: CD63, CD81, CD82 and CD9. The number of exosomes released by the cell can be regulated by the lysosomal degradation. In this situation, the MVB and a lysosome fuse together, degrading the MVB’s content. Rab27a/b GTPases regulate cellular vesicle trafficking. Microtubules guide the MVB to the cell membrane. Actin cytoskeleton also helps in the MVB docking. (2) The SNARE complex is responsible for the blending of plasma membrane with the MVB. When exosomes are delivered into the extracellular medium, they reach out target cells by using MHC and other transmembrane proteins and receptors. Exosomes can transport and transfer a huge variety of exosomal cargoes. (3) Microvesicle biogenesis follows a different pathway: budding of the plasma membrane produces microvesicles. hnRNPA2/B1: heterogeneous nuclear ribonucleoproteins A2/B1; ESCRT: endosomal-sorting complex required for transport; MVB: Multivesicular Body; ILV: Intraluminal vesicle; SNARE: soluble N-ethylmaleimide-sensitive fusion attachment protein receptor; MHC: major histocompatibility complex; MMP: matrix metalloproteinase; ADAM: a disintegrin and metalloproteinase

Exosome biogenesis consists of multiple steps, which are highly regulated by intracellular and extracellular signals. Initially, an early endosome derived from the plasma membrane is formed. The subsequently inward budding of the early endosome generates intraluminal vesicles (ILVs), leading to the formation of a MVB [Figure 1].

Exosomes can transport a huge diversity of functional molecules: that go from lipids to genetic material. One of the main stages in exosome formation is the sorting of its cargo. It is known that molecules taking part in exosome biogenesis first bring exosomal cargoes really close to the membrane of the MVB in order to load them into exosomes^[20]^. In the case of RNA loading, a group of RNA binding proteins, the heterogeneous nuclear ribonucleoproteins (hnRNPs), have been shown to play a crucial role. This family of proteins is important for the transcription, splicing, transport and maturation of RNA^[37]^. One of the members, hnRNPA2B1, selectively binds to certain miRNAs directing their loading into exosomes^[38]^. Despite this, very little is known about the regulation of this process and other intracellular RNA transport mechanisms, which could play an important role^[39]^.

In early MVBs, exosomes can be formed in the following ways (1) an endosomal-sorting complex required for transport (ESCRT)-dependent^[40]^ or (2) ESCRT-independent pathway. The ESCRT is a cluster of four different subunits (0-III) that drives exosome shaping and MVB generation. ESCRT-0 and ESCRT-I ubiquitinylate exosomal cargoes are located on the membrane and activate ESCRT-II, recruiting ESCRT-III, which is responsible for membrane budding^[41]^. The ESCRT-independent pathway for exosome biogenesis depends on the generation of ceramide. Neutral Sphingomyelinase type II (nSMase2) is the enzyme that produces ceramide by hydrolyzing sphingomyelin located in the MVB membrane. In fact, several studies have shown that the inhibition of nSMase2 by using GW4869 reduces exosome biogenesis and the release of exosomes^[42]^.

On the exosome surface several transmembrane proteins belonging to the tetraspanin family can be found. Particularly, CD63 is highly enriched on late endosomes, MVBs and exosomes, participating in endosomal sorting in both, ESCRT-dependent/independent pathways, and being a useful biomarker for exosome detection^[43]^. Other tetraspanins, such as CD81, CD82 and CD9 are involved in cargo loading into exosomes^[20]^ .

Once the MVB has maturated, the cytoskeleton transports it towards the plasma membrane^[23]^. Both, actin and microtubules orchestrate MVB motility. Rab GTPases proteins, which control vesicular trafficking, allow the docking of the MVB and the release of exosomes into the extracellular space. More than 70 molecules form the Rab GTPases family exist^[44]^. Among these, Rab27a and Rab27b have been shown to specifically command the docking of MVBs. In fact, by knocking down Rab27a/b using an short hairpin RNA (shRNA), exosome production was shown to reduce significantly^[45]^. Rab27a/b GTPases are responsible of the first stages in exosomal transport, however the SNARE is in charge of MVB fusion with the cellular membrane, and thus exosome release to the medium^[46]^.

Although some of the mechanisms and molecules underlying exosomal biogenesis are already established, there is still a lack of knowledge regarding the involvement of other molecules in exosome regulation and production in cancer. Additionally, it has been shown that acidic pH directly increases exosome release in cancer^[47]^. Meaning that, the local tumor microenvironment can control the number of exosomes released by the cells, regulating cellular communication.

### Exosome-induced drug resistance

Up to date, exosomes have been shown to induce pharmacological desensitization by different mechanisms. One of the most well studied mechanisms is by protein transfer. ATP-dependent multidrug transporter P-glycoprotein (Pgp) expels drugs located in the cytoplasm to the extracellular media, inducing drug resistance^[48]^. Exosomes transporting Pgp, fusion with osteosarcoma cells plasma membrane, enriching the tumor cell with Pgp transporters and causing pharmacologic desensitization^[49]^. In addition, serum detection of exosomes carrying Pgp has been reported to be a potential biomarker to identify docetaxel resistance in prostate cancer patients^[50]^.

Exosomes have also been shown to act against immunotherapeutics operating as fake targets. The main treatment for aggressive B-cell lymphoma is Rituximab, nevertheless some patients develop resistance to this drug. Lately, it has been reported that exosomes secreted by lymphoma B-cells, were enriched with CD20. Thus, the monoclonal antibody anti-CD20, Rituximab, was incorrectly binding to the decoy exosomal-CD20, instead of the cellular-CD20, generating resistance to this immunotherapeutic drug^[51]^.

Another system to induce drug resistant phenotypes through exosomes is the transmission of nucleic acids (mRNA, ncRNA, *etc*). Because lncRNAs are the least discussed ncRNA cargos of exosome, in the following sections will go into detail about exosomal lncRNAs in cancer.

## EXOSOMAL NCRNAS

Especially interesting is the tight relationship between exosomes and ncRNAs. Valadi *et al.*^[52]^ in 2007 described, for the first time, the intercellular transferring of RNAs, specifically mRNAs and miRNAs, through exosomes. His work sprouted research on exosomal ncRNAs as functional effectors in cellular communication.

The human genome is mainly composed of non-coding elements and, particularly, more than half of the non-coding transcriptome are transcribed ncRNAs^[53]^. The functions of these non-coding regions of our genome are still debated. Nevertheless, there is increasing data about ncRNAs’ implication in cancer^[54]^.

MiRNAs are the most deeply studied ncRNA^[55,56]^. Several lines of evidence suggest that miRNAs act as hormone-like molecules and are dysregulated in cancer^[57,58]^. Donor cells can release miRNAs directly into the extracellular space associated with AGO (argonaute), inside exosomes or bound to lipids. Extracellular miRNAs can be found in all biological fluids and are potential biomarkers for detecting a variety of diseases, including cancer^[59]^. In breast cancer patients, miR-21 and miR-1246 were detected to be increased in exosomes from plasma compared to healthy patients. Proving that exosomal extracellular miRNAs can be employed as biomarkers for spotting breast cancer^[60]^. In addition, several studies have reported that exosomal miRNAs are captured by recipient cells and regulate their gene expression, changing cell phenotype and for example, promoting tumorigenesis^[61]^. Moreover, exosomal miRNAs induce drug resistance, for example; in lung cancer, miR-i00-5p developed cisplatin desensitization^[62]^. Many other reviews have summarized the importance of exosomal miRNAs in cancer drug resistance^[63,64]^, but the roles of exosomal lncRNAs in drug resistence were only marginally systematized.

## ROLES OF EXOSOMAL LNCRNAS

LncRNAs are RNA molecules longer than 200 ribonucleotides, which are differentially expressed in cancer^[54]^. The resemblance between mRNA and lncRNAs has been reported in several occasions^[65]^: They can be transcribed with RNA-polymerase II from different DNA loci and suffer post-transcriptional modifications, such as poly-adenylation. In contrast to mRNAs, lncRNAs, have little or no translation potential^[66]^. LncRNAs can execute several functions working as scaffolds, guidance molecules, signaling pathways, and be decoys for other molecules^[67–69]^. Additionally, these long RNA molecules regulate gene expression by recruiting, to a specific DNA region, epigenetics modifiers or chromatin-remodelling complexes^[66]^. One example is the renowned lncRNA XIST, responsible for the inactivation of one of the two sexual X chromosomes in female embryos^[70]^. LncRNAs also conduct transcriptional regulation by summoning transcription factors and activating^[71]^ or repressing RNA polymerase II^[72]^. Furthermore, lncRNA control post-trancriptional stages acting as “sponges” by physically interacting and capturing miRNAs. Consequently, these molecules are named: competing endogenous RNAs (ceRNAs)^[73]^. In colon cancer, SNHG7 lncRNA, was recently reported to sponge miR-2i6b enhancing pro-tumoral activity and metastasis^[74]^. LncRNAs can regulate gene expression in an immense variety of different regulatory systems. These data prove the huge heterogeneity of lncRNAs. All these is possible because lncRNAs have a complex natural structure, comprising double helices or hairpin loops, which provide the versatility of lncRNAs to interact with distinct molecules.

Among all ncRNAs, lncRNAs have also been recently found in exosomes and associated with cancer. Similarly to miRNAs, lncRNAs proportion differs between cells/exosomes and healthy/pathologic conditions^[75]^. Tumor liquid biopsies have emerged as a non-invasive system to detect potential biomarkers for cancer prognosis, diagnosis and evolution. One of the major components of liquid biopsies are circulating lncRNAs, which can be found freely or encapsulated in exosomes. For instance, PCA3 is the only FDA-approved ncRNA that can be detected in urine and has a diagnosis potential in prostate cancer^[76,77]^. Specifically, exosomal lncRNAs have attracted scientific community attention because of their stability and preservation in several bodily fluids besides blood, such as: saliva, urine, *etc.*^[78]^. In serum from non-small cell lung cancer (NSCLC) patients, exosomal MALAT-1 (metastasis associated lung adenocarcinoma transcript 1) was higher compared to healthy patients^[79]^. In contract, serum exosomal GAS5 was down-regulated in NSCLC patients and negatively correlated with tumor stages^[80]^. Other research group, reported that exosomes containing SOX2-OT (Sox2 overlapping transcript) are enriched in plasma from patients with lung squamous cell carcinoma^[81]^. Taking everything into consideration, exosomal lncRNAs could be used as a solution for the early detection of cancer and can become a priceless diagnostic tool^[17]^.

**Figure 2.**
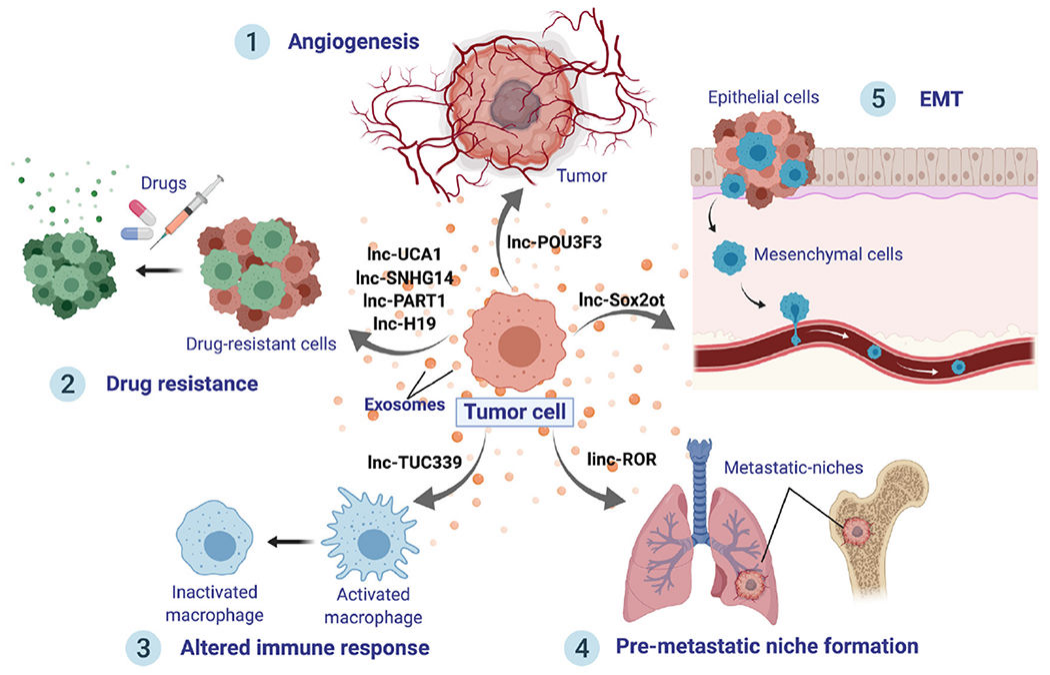
Roles of exosomal lncRNAs. Exosomes from malignant cells are released to the cancer microenvironment and distant organs, delivering lncRNAs, which can induce tumor progression. LncRNAs can exert several effects onto recipient cells: (1) Promote angiogenesis, such as lnc-POU3F3; (2) Boost cancer drug resistance; (3) Shape immune cell signaling, interfering and altering the immune response; (4) Stimulate the creation of a distant pre-metastatic niche; and (5) Trigger epithelial-mesenchymal transition, which will, in turn, hasten metastases

Mechanistically, exosomal lncRNAs are essential players in cellular communication, allowing tumor cell progression and metastasis [Figure 2]. In order to supply enough nutrients, one of the main factors for enhancing tumor promotion is angiogenesis. Following this line, angiogenesis was shown to be promoted by exosomal secretion of lncRNA POU3F3 (lncRNA POU class 3 homeobox 3)^[82]^. Overall, lncRNAs demonstrate to be essential molecules to change endothelial cell phenotypes, using exosomes as delivery packages.

Furthermore, exosomal lncRNAs have been found to boost metastasis by stimulating EMT and PMN formation. For instance, exosomal transfer of lnc-Sox2ot was shown to trigger EMT^[83]^. Additionally, linc-ROR contained into exosomes has been reported to promote EMT and reinforce the establishment of a distant PMN^[84]^. The immune system is a fundamental element in cancer progression. Lately, the transfer of lncRNA TUC339 from hepatocellular carcinoma (HCC) exosomes was shown to inactivate macrophages, suppressing cytokine production and phagocytosis^[85]^. Hence, exosomal lncRNAs shape the immune system resulting in an altered immune response in cancer. Moreover, exosomal lncRNAs have been described to exert a decisive role in cancer drug resistance. This subject will be covered extensively in the following sections.

## EXOSOMAL LNCRNAS PROMOTE DRUG RESISTANCE

In primary localized tumors, the most effective treatments are radiotherapy and surgical extirpation. Nevertheless, in order to avoid recurrence or when metastasis occurs, treatments involving drugs are essential. Nowadays, cancer drug therapy is mainly classified in four categories according to the molecular target and mechanism of action of each group: (1) chemotherapy;(2) targeted therapy; (3) hormone therapy; and (4) immunotherapy. Several articles support the idea that transferring exosomal lncRNA may change target-cells phenotypes and its response to treatment [Table 1]. The implication of exosomal lncRNAs in promoting drug resistance will be addressed in this section.

### Chemotherapy

Chemotherapeutic drugs imprecisely inhibit cell proliferation not only affecting cancer cells growth, but also non-neoplastic cells, leading to a huge toxicity and secondary effects^[86]^. Temozolomide (TMZ) is an alkylating drug employed as a regular treatment in glioblastoma multiforme (GBM). Many patients become resistant to TMZ, however the molecular mechanisms underlying TMZ acquired desensitization are poorly understood. Recently, Zhang *et al.*^[87]^ demonstrated that exosomal lncRNA-SBF2 antisense RNAi (lncRNA SBF2-AS1) promoted TMZ resistance. They used GBM-TMZ resistant and sensitive cell lines to understand the mechanism. By fluorescence in situ hybridization (FISH) and real-time reverse transcription PCR (qRT-PCR) they showed that TMZ-resistant tissues and cell lines expressed higher SBF2-AS1 compared to the sensitive ones. LncRNA SBF2-AS1 overexpression in TMZ-sensitive cell lines reversed the chemotherapeutic effect of TMZ, conferring TMZ-resistance. In contrast, knocking-down lncSBF2-AS1 contributed to TMZ-sensitivity. Exosome purification and characterization revealed high levels of SBF2-AS1 and clinically TMZ-resistant patients exhibited increased levels of enriched-SBF2-AS1 exosomes in serum. Moreover, by western blot, immunofluorescence and RNA immunoprecipitation, SBF2-AS1 was reported to sponge miR-i5ia-3p up regulating the expression of X-ray repair cross-complementing 4 (XRCC4) protein. XRCC4 is responsible of double stranded DNA repair and thus, promotes cell survival and cell invulnerability to TMZ-cytotoxicity.

Linc-VLDLR is a lncRNA that has an important function in inducing resistance to chemotherapeutic drugs (camptothecin and doxorubicin) and sorafenib targeted therapy in HCC. After exposure to camptothecin, doxorubicin and sorafenib, HCC cell lines and derived-exosomes expressed high levels of linc-VLDLR. Interestingly, incubation with linc-VLDLR-enriched EVs, reduced apoptosis. In order to elucidate the molecular mechanisms by which linc-VLDLR induces drug resistance, the lncRNA was knocked-down by interference RNA: consequently, ATP-binding cassette, sub-family G member 2 (ABCG2), a drug ejecting protein, was down-regulated^[88]^. The same research team reported another lncRNA, linc-ROR, to contribute in doxorubicin and sorafenib resistance in HCC. Several HCC cell lines were exposed to TFG β (Transforming Growth factor β) and/or doxorubicin-sorafenib. As a result, exosomes presented higher linc-ROR content and were resistant to chemotherapy. HCC cells were then co-cultured with exosomes enriched in linc-ROR, resulting in increased survival after drug treatment. Altogether these data confirm the role of exosomal linc-ROR inducing drug resistance in HCC. Furthermore, knocking-down linc-ROR, increased HCC cells apoptosis when being treated with doxorubicin and sorafenib. In addition, caspase 3/7, was found to be increased^[89]^. Overall, linc-VDLDR and linc-ROR, were reported to actively participate in exosome-mediated drug resistance.

### Targeted Therapy

In order to avoid the toxic effects of chemotherapeutic drugs, targeted therapy emerged as the perfect answer. Advances in molecular biology, allowed the identification of molecular pathways involved in cancer. Consequently, researchers elaborated specific drugs to target these molecules implicated in cancer promotion^[86]^. As already mentioned in the previous section, linc-VLDLR and linc-ROR were shown to promote resistance in HCC to the targeted therapeutic, sorafenib.

Recently, Dong *et al.*^[90]^ demonstrated that the lncRNA-small nucleolar RNA host gene 14 (SNHG14) is overexpressed in trastuzumab-resistant HER2+ breast cancer cells in comparison to sensitive ones. The same outcome was found in exosomes derived from resistant cells. The SNHG14-enriched-exosomes were co-cultured with sensitive HER2+ breast cancer cell lines, resulting in a trastuzumab-desensitizing phenotype. The effect was reversed by knocking-down SNHG14. The molecular mechanisms by which lncRNA SNHG14 generates trastuzumab resistance are not completely unraveled, however a signal transduction reporter array pointed out the role of the apoptosis regulator pathway Bcl-2/Bax.

Cetuximab is a monoclonal antibody employed in colorectal cancer (CRC) that binds to EGFR (epidermal growth factor receptor) inducing its degradation. However, many tumors are resistant to cetuximab. Thus, predictive biomarkers for cetuximab resistance would be useful for treatment selection. In this context, UCA1 was shown to be overexpressed in resistant CRC cells compared to parental cells and promises to be a novel clinical biomarker for cetuximab resistance^[91]^.

Prostate Androgen-Regulated transcript 1 (PART 1) is a lncRNA that provokes gefitinib desensitization in esophageal squamous cell carcinoma. Gefitinib is a targeted drug therapy that inhibits multiple tyrosine kinases. PART1 was reported to be increased in gefitinib-resistant cells. By FISH, extracellular PART1 was found to be enclosed in exosomes. *In vivo* nude mice xenografts injected with PART1-transfected TE1 cells, showed that PART1-TE1 tumors grew significantly faster compared to the controls, while treated with gefitinib. The molecular mechanism of PART1-induced gefitinib-resistance was also described: PART1 competitively sponges miR-129, consequently Bcl-2 mRNA is no longer inhibited, increasing the expression of its protein and reducing apoptosis. Exosomal PART1 could be used in the clinic as a treatment selection biomarker. High PART1 expression in serum exosomes indicates gefitinib resistance^[92]^. Another exosomal lncRNA, H19, has also been reported to promote gefitinib resistance. Following similar methods as the ones previously described, researchers demonstrated that H19 packed into exosomes induces gefitinib-desensitization in NSCLC. As a result exosomal H19 could be used as a molecular biomarker to detect gefitinib-resistance in NSCLC^[93]^.

Erlotinib, along with gefitinib, is another tyrosin kinase inhibitor. Resistance to erlotinib is a frequent obstacle in NSCLC therapy. For this reason, the role of several exosomal lncRNAs in erlotinib resistance have been studied to unravel possible implicated molecules. Recently, the lncRNA RP11-838N2.4 has been identified to be up-regulated in erlotinib-resitant NSCLC cells. FOXO1 could be regulating the expression of this lncRNA by recruiting histone deacetylases to its promoter region. Moreover, exosomes in erlotinib-resistant NSCLC patients were highly enriched with RP11-838N2.4^[94]^.

Qu *et al.*^[25]^ identified for the first time the lncARSR [lncRNA-Activated in renal cell carcinoma (RCC) with Sunitinib Reistance] in RCC. LncARSR overexpression was linked to poor response to sunitinib treatment, a tyrosine kinase inhibitor. In addition, lncARSR exosomal transfer induces sunitinib desensitization in the recipient cell. They also demonstrated the molecular mechanisms by which lncARSR promotes sunitinib resistance. Shortly, the lncRNA binds to mir-34 and miR-449. This physical interaction sponges both miRNAs and increases the expression of their downstream targets: c-MET and AXL which are accountable for sunitinib-resistance.

### Hormone therapy

Hormone therapy is mainly based on molecules that tackle hormone synthesis or activity in hormone-dependent cancers. The vast majority (75%) of breast cancers are positive for hormonal receptors^[95,96]^. These molecular subtypes are sensible to endocrine therapy, thus the ER blocker Tamoxifen, is considered the first-line hormonal treatment for estrogen receptor positive (ER+) breast cancer^[97,98]^. Xu *et al.*^[99]^ described the lncRNA Urothelial cancer associated 1 (UCA1) as an exosomal transmitter of tamoxifen drug resistance in breast cancer. Exosomes derived from tamoxifen-resistant breast cancer cells, LCC2, exhibited greater expression of UCA1 compared to sensitive cells (MCF7). MCF7 tamoxifen-sensitive cells were treated with LCC2-derived exosomes (high UCA1 content) resulting in decreased apoptosis after tamoxifen treatment. In conclusion, exosomal UCA1 can be transferred from tamoxifen-resistant cells to responsive-cells, inducing drug resistance.

### Immunotherapy

Immune checkpoint blockade is the most researched immunotherapy. Immune checkpoints consist of multiple surface molecules that maintain immune equilibrium and prevent autoimmune reactions. In cancer, immune checkpoints enable the immune evasion of cancer cells. Tumor cells undergo several transformations acquiring reduced antigenicity by enhancing immunoinhibitory molecules and attracting immunosuppressive cells to the local tumor microenvironment^[100]^. The first (food and drug administration) approved immune checkpoint inhibitors are targeting programmed cell death 1 (PD-1) or programmed cell death ligand 1 (PD-L1). These immunotherapeutic drugs block PD-1 or PD-L1, enhancing the immune response against the tumor. For instance, the PD-1 antibodies, nivolumab and pembrolizumab, approved by the FDA in 2014 and 2017, respectively, are indicated for advanced metastatic melanoma, CRC, NSCLC, RCC, castration-resistant prostate cancer and other solid resistant tumors, such as triple-negative breast cancer (TNBC)^[101]^. TNBC represents 20% of all breast cancers. The lack of expression of hormonal receptors excludes the usage of hormonal therapy and targeted therapy, leaving chemotherapy as the only treatment option. The development of immunotherapeutic drugs, such as pembrolizumab, opened new treatment approaches for TNBC. However, many TNBC patients are resistant to PD-1 blockade drugs^[102]^. In these patients, the lncRNA LINK-A, was recently shown to be upregulated. Moreover, LINK-1 was reported to mediate the degradation of the antigen peptide-loading complex (PLC). Consequently, LINK-1 decreases antigenicity and promotes PD-1 immune checkpoint inhibitor resistance^[103]^. It has not yet been reported if LINK-A can be transferred through exosomes in order to transmit PD-1 inhibitors resistance. Thus, research should also be focused on this direction.

## CONCLUSION

Nowadays, one of the main challenges in oncology is drug resistance. Tumor microenvironment, along with cancer cells, are implicated in pharmacologic resistance development^[6,7,34]^. Consequently, non-neoplastic stroma cells should be taken into consideration when assessing tumor status and treatment options^[11]^. Moreover, all cells constantly communicate utilizing several systems. Specifically, exosomes have been shown to exert an essential role in drug desensitization by transferring proteins, mRNAs, or ncRNAs from donor cells to recipient cells^[18,19,52]^.

Up to date, a huge variety of exosomal lncRNAs have been shown to induce cancer drug resistance. Interestingly, the vast majority of these drugs are targeted therapy molecules. This might indicate that the more specific is the molecule, the more likely is drug resistance going to occur.

Although the molecules involved in the biogenesis of exosomes have been studied, the extracellular and intracellular signals regulating this process still need to be unraveled. Additionally, the molecular mechanisms by which lncRNAs are loaded into exosomes have not been completely elucidated. Moreover, it would be crucial to research the mechanism by which each drug activates exosome biogenesis and the specific lncRNA sorting into exosomes.

The lncRNA LINK-1 induces PD-1 drug resistance. Nevertheless, no articles report these lncRNAs to be packaged into exosomes^[103]^. Consequently, it should be considered to study if LINK-1 induced-drug can be transferred to sensitive cells through exosomes.

In addition, identifying the exosomal lncRNAs involved in drug resistance could be a useful strategy to develop drug resistance/response biomarkers in tumor liquid biopsy. Finally, knowing the mechanisms by which lncRNAs induce drug resistance could lead to the development of new potent drugs or therapeutic strategies that could reverse drug desensitization.

## Figures and Tables

**Table 1.** Exosomal lncRNAs involved in drug resistance

lncRNA	Cancer type	Cell line	Drug	Mechanism	Ref.
UCA 1	BC (Estrogen receptor positive)	MCF-7, LCC2	Tamoxifen (HT)	Not stated	Xu *et al.*^[99]^
SNHG14	BC [Human epidermal growth factor receptor 2 (HER2) positive]	SKBR-2, BT474	Trastuzumab (TT)	Targets Bcl-2 and Bax apoptosis pathway	Dong *et al.*^[90]^
UCA1	CRC	Caco2	Cetuximab (TT)	Not stated	Yang *et al.*^[91]^
PART1	Esophageal squamous cell carcinoma	TE1, KYSE-450	Gefitinib (TT)	Competitive binding to miR-129 increasing Bcl-2 expression.	Kang *et al.*^[92]^
SBF2-AS1	GBM	U87,LN229, A172, T98, U251	Temozolomide (CT)	Acts as a ceRNA for miR-151a-3p, desinhibits XRCC4 target, reparing DSB.	Zhang *et al.*^[87]^
Linc-VLDLR	HCC	MzChA-1, Huh-7, HepG2, PLC-PRF-5, Hep3B	Several drugs: sorafenib (TT), camptothecin (CT), doxorubicin (CT)	ABCG2	Takahashi *et al.*^[88]^
Linc-ROR	HCC	HepG2, PLC-PRF5	Several drugs: sorafenib (TT), doxorubicin (CT)	TGFβ enriches linc-ROR in extracelular vesicles.	Takahashi *et al.*^[89]^
H19	NSCLC	HCC827, HCC4006	Gefitinib (TT)	Not stated	Lei *et al.*^[93]^
RP11-838N2.4	NSCLC	HCC827, HCC4006	Erlotinib (TT)	This lncRNA is negatively regulated by FOXO1	Zhang *et al.*^[94]^
lncARSR	RCC	ACSu3rd, 7Su3rd.	Sunitinib (TT)	Facilitate AXL and c-MET expression by competitive binding to miR-34/miR-449.	Qu *et al.*^[25]^

CT: Chemotherapy; TT: targeted therapy; HT: hormone therapy; BC: breast cancer; CRC: colorectal cancer; GBM: glioblastoma multiforme; HCC: hepatocellular carcinoma; NSCLC: non-small cell lung cancer; RCC: renal cell carcinoma; ABCG2: ATP-binding cassette, sub-family G member 2; TGFβ: Transforming growth factor β; DSB: double-strand breaks
